# Relationship of regulatory T cells to *Plasmodium falciparum* malaria symptomatology in a hypoendemic region

**DOI:** 10.1186/1475-2875-13-108

**Published:** 2014-03-19

**Authors:** Katherine J Torres, Elizabeth Villasis, Jorge Bendezú, José Chauca, Joseph M Vinetz, Dionicia Gamboa

**Affiliations:** 1 Instituto de Medicina Tropical “Alexander Von Humboldt”, Universidad Peruana, Cayetano Heredia, AP 4314, Lima 100, Peru; 2 Departamento de Ciencias Celulares y Moleculares, Laboratorios de Investigación y Desarrollo,Facultad de Ciencias y Filosofía, Universidad Peruana Cayetano Heredia, AP 4314, Lima 100, Peru; 3 Division of Infectious Diseases, Department of Medicine, University of California San Diego, La Jolla, CA, USA

**Keywords:** Regulatory T cells, Asymptomatic, Symptomatic

## Abstract

**Background:**

Previous data have suggested that regulatory T cells (Tregs) balance protective immune responses with immune mediated pathology in malaria. This study aimed to determine to test the hypothesis that Treg proportions or absolute levels are associated with parasitaemia and malaria symptoms.

**Methods:**

Treg cells were quantified by flow cytometry as CD4+ CD25+, Foxp3+, CD127^low^ T cells. Three patient groups were assessed: patients with symptomatic *Plasmodium falciparum* malaria (S), subjects with asymptomatic *P. falciparum* parasitaemia (AS) and uninfected control individuals (C).

**Results:**

S, AS and C groups had similar absolute numbers and percentage of Tregs (3.9%, 3.5% and 3.5% respectively). Levels of parasitaemia were not associated with Treg percentage (p = 0.47).

**Conclusion:**

Neither relative nor absolute regulatory T cell numbers were found to be associated with malaria-related symptomatology in this study. Immune mechanisms other than Tregs are likely to be responsible for the state of asymptomatic *P. falciparum* parasitaemia in the Peruvian Amazon; but further study to explore these mechanisms is needed.

## Background

*Plasmodium falciparum*-infected individuals show a wide spectrum of clinical manifestations that range from a state of asymptomatic infection to severe life-threatening forms, such as hyperparasitaemia, hypoglycaemia, cerebral malaria, respiratory distress, and vital organ dysfunction [1,2]. In recent years, the concept of asymptomatic parasitaemia and its potential interaction with the human immune system to malaria infection has emerged on the forefront of why some individuals are able to control the infection with lack of symptoms [3-6]. This concept implies that individuals with low parasitaemia, in the absence of clinical symptoms (i.e. fever, headache, chills, malaise, etc.) evidence that down-regulation simultaneously of both the innate immune system and antigen-specific dependent immunity is obvious [3,5,6].

Elevated levels of circulating tumor necrosis factor (TNF), interleukin-6 (IL-6), IL-12, IL-1β, and IL-10 have been reported to correlate with malaria disease severity and with fatal outcomes [7,8]; the behaviour of these cytokines in asymptomatic parasitaemia remains unknown. Regardless, the mechanisms by which acquired and innate immune mechanisms lead to the state of asymptomatic malaria parasitaemia remain unexplored.

That much of the symptomatology of malaria infection seems to be immune-mediated implies that protective *versus* pathological immune responses are tightly regulated. Interest has recently arisen in mechanisms of immune regulation mediated by CD4^+^, CD25^+^, FoxP3^+^ regulatory T cells (Tregs). This T-cell subset has been shown to play an important role in maintaining immune homeostasis and controlling excessive immune responses [7]. These cells have been reported to suppress cellular immune responses through direct contact with immune effectors cells and by production of regulatory cytokines, including TGF-ß and IL-10 [7].

Previous work has shown that Tregs significantly modify the host response to malarial infection including modulation of responses to *P. falciparum* pro-inflammatory moieties such as GPI anchors and DNA-adsorbed to haemozoin [7,9], and self-regulation moderated by the broader immunological environment. In turn, the host response to malarial infection is influenced by both the genetic background of the host and co-infection with other pathogens [9,10]. Tregs have been reported to modify blood-stage infection *in vivo* in humans including higher parasite growth rates [10-12] and elevated production of transforming growth factor-beta (TGF- β) and IL-10 [11] that lead to down-modulation of inflammatory responses mediated through interferon-gamma (IFN-γ) [5,11].

In the low transmission setting of Amazonia, asymptomatic malaria parasitaemia is surprisingly common [3,6,11,13,14]. This paradoxical pattern stands in contrast to high transmission regions where acquired immunity is manifested by asymptomatic parasitaemia that typically takes years and intense seasonal or continuous transmission to develop. Important exceptions have been demonstrated when malaria-naïve migrants move as adults to high transmission regions [13]. Hence, there is an abundance of asymptomatic malaria infection, the functional consequences of which remain unclear and relatively neglected. In this particular study in general, the hypothesis that the state of asymptomatic malaria parasite involves both antigen-specific acquired immunity and down-regulation of pro-inflammatory innate immune responses that may promote the ability of asymptomatic reservoirs to transmit infection to vector mosquitoes.

Understanding mechanisms of clinical immunity manifested by asymptomatic malaria parasitaemia has emerged as a major issue in the malaria field [13]. This study aimed to test the specific hypothesis that absolute numbers of circulating Tregs contribute to maintain a state of asymptomatic malaria parasitaemia. Tregs in symptomatic and asymptomatic subjects with *P. falciparum* malaria-hypoendemic region of the Peruvian Amazon were studied.

## Methods

### Study site and population

The study was carried out in communities near Iquitos, Peru in the Amazon region. Strategies for identifying patients were as follows:

A. *Passive case detection* (PDC), where samples were obtained from presenting to the San Juan Bautista Health Centre in Iquitos. All samples obtained here came from symptomatic patients with uncomplicated malaria. The patients attended the Health Centre because of self-reported fever. *Plasmodium falciparum* infection was confirmed after light microscopic examination of stained thick and thin peripheral blood smears, and later further confirmed at the species level using a specific PCR assay [15]. Health post personnel were accurate in accurately identifying the presence of *P. falciparum*.

B. *Active case detection* (ACD), the sampling of asymptomatic parasitaemic individuals, was based on identifying collaterals. Once the identification of a symptomatic subject with *P. falciparum* infection during passive case detection, the index case’s family and neighbours were visited weekly over the subsequent month to identify additional asymptomatic cases, assuming local transmission and acquisition of infection.

Furthermore, there was an ACD survey carried out in the small village of Atalaya located 6–7 hours northwest of Iquitos by motor-boat. This area was chosen as a study site because of reports of an outbreak of *P. falciparum* in March of 2010. 14 samples from symptomatic/asymptomatic individuals were obtained in this community.

Whole blood samples were collected from 26 patients with *P. falciparum* malaria, 15 with acute uncomplicated malaria (S), 11 with asymptomatic malaria (AS) and seven adults who denied a history of malaria (Controls, C). The diagnosis of *P. falciparum* malaria infection was based on the light microscopy examination of Giemsa-stained thick blood films. Real time polymerase chain reaction (PCR) with species-specific primers was performed on DNA isolated from dry blood samples on filter paper to verify malaria infection [15]; blood cell counts were performed using Turk’s solution. All subjects had *P. falciparum* mono-infection; no subject had *P. vivax* infection identified either by peripheral blood smear or PCR.

### Ethical consent

The study was approved by the Ethics Committee of Universidad Peruana Cayetano Heredia, Peru. All subjects provided written informed consent prior to enrollment.

### Flow cytometric analysis

Unprocessed whole blood was shipped at ambient temperature the same day from Iquitos to Lima and processed within 8 hr of blood draw. PBMCs were isolated from heparinized blood by density gradient centrifugation (BD Vacutainer CPT Cell Preparation Tube with sodium heparin, NJ). Regulatory cells were defined as being CD4^+^, CD25^+^, CD127^low^ and FoxP3 by flow cytometry. PBMCs were first stained using peridinin-chlorophyll-a (PerCP)- conjugated anti-CD4 and phycoerythrine (PE)-conjugated anti-CD25 monoclonal antibodies (BD Biosciences, San Jose, California, USA). After fixing and permeabilizing, the cells were then stained for intracellular FoxP3 using a fluorescein-isothiocyanate (FITC)-conjugated anti-FoxP3 monoclonal antibody (eBiosciences, San Diego, California, USA). Cells were analysed using a FACScalibur flow cytometer (Beckton Dickinson, Franklin Lakes, New Jersey, USA). Regulatory T cells were identified as CD25+ and FoxP3 + cells among CD4+ cells within the lymphocyte gate. Absolute CD4+ cell counts were performed using a 4 color single platform staining of whole blood cells (anti CD3-FITC, CD4-PE, CD45 PerCP and CD8 APC). Flow cytometry analysis used FlowJo software (V.8.5 Tree Star, Ashland, Oregon, USA).

### Statistical analysis

The non-parametric Kruskal-Wallis test was used to compare the values of each variable among different groups. The Mann–Whitney *U* test with the Bonferroni correction was used for post-hoc comparisons. The linear relationship between Treg cell percentage and peripheral parasitaemia was measured by Spearman’s Rank Correlation Coefficient. The level of significance was set at a P value of < 0.05 for all analyses.

## Results

A significant decrease in the number of circulating lymphocytes in S individuals (Table 1) was found to be related to *P. falciparum,* and could be related to migration to lymph nodes during acute infection as reported previously [16]. AS individuals infected with *P. falciparum* had more circulating leukocytes compared to S individuals (5.7 × 10^9^/L and 5.0 × 10^9^/L) respectively; *p* = 0.002. In contrast, S individuals had total leukocytes compared to C individuals (1.41 × 10^9^/L and 2.0 10^9^/L) respectively; *p* = 0.005. Nonetheless, total lymphocyte counts were similar between S and AS individuals (1.41 × 10^9^/L and 1.78 × 10^9^/L, respectively). The levels of parasitaemia were significantly different in S compared to AS individuals *p* = 0.0003 (Table 1).

**Table 1:** Demographic and clinical characteristics of study participants

**Plasmodium falciparum infection status**						
**Characteristics**	**Symptomatic**	**Asymptomatic**	**Control**	**P value**		
	**(S)**	**(AS)**	**(C)**	**(C vs. S)**	**(C vs. AS)**	**(S vs. AS)**
No. of subjects	n = 15	n = 11	n = 7			
Gender (% male)	60.00%	55.55%	71.43%			
Median age (year) [range]	28 [9–59]	17 [12–41]	30 [22–34]	0.589	0.389	0.42
No. of Leukocytes (10^9^/L)*	5.0 [4.3–6.0]	5.7 [5.1–7.6]	5.5 [4.2–6.5]	0.203	0.276	0.002
No. of Lymphocytes (10^9^/L)*	1.41 [0.93–2.1]	1.78 [1.28–2.58]	2.0 [1.4–2.6]	0.0054	0.389	0.018
No. of Tregs (10^6^/L)*	7.88 [3.38–24.08]	8.66 [5.01–16.06]	11.09 [8.88–11.62]	0.29	0.297	0.979
Parasitaemia**	5999 [71–52,900]	1 [1–2874]	NA	NA	NA	0.0003

*Median [range] and means, the value of 50 percentile [min – max].

**N° of parasites ml^-1^.

*P* values are from Mann–Whitney *U* test (to compare two non-parametric group distributions by rank).

The proportion of male gender was compared by Chi-square test. Only for gender the test was the Chi-square test used.

**Figure 1:**
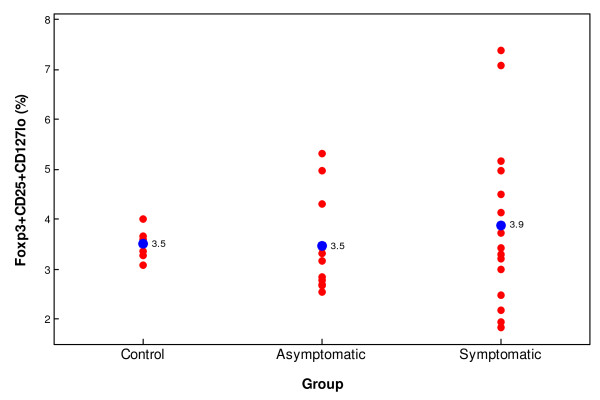
**Percentage of Tregs in control, symptomatic and asymptomatic subjects.** The blue dots indicates the median. No statistically significant differences were found among groups.

Treg cells identified by flow cytometry as CD4^+^ T cells expressing Foxp3, CD25^+^ and low levels of CD127^low^ were measured and reported as a percentage of total CD4^+^ T cells. There were no significant differences in Treg cell percentage among patients with uncomplicated *P. falciparum* malaria S (median 3.9%), AS (median 3.5%), and C (median 3.5%) individuals [*H* = 0.613, df = 2, *p* = 0.736] (Figure 1), or absolute Treg cell numbers between S and AS patients (median 7.88 × 10^6^/L [3.38–24.08 × 10^6^/L] and median 8.66 [5.01–16.06 × 10^6^/L], respectively) (Table 1), according to the Kruskal-Wallis test.

**Figure 2:**
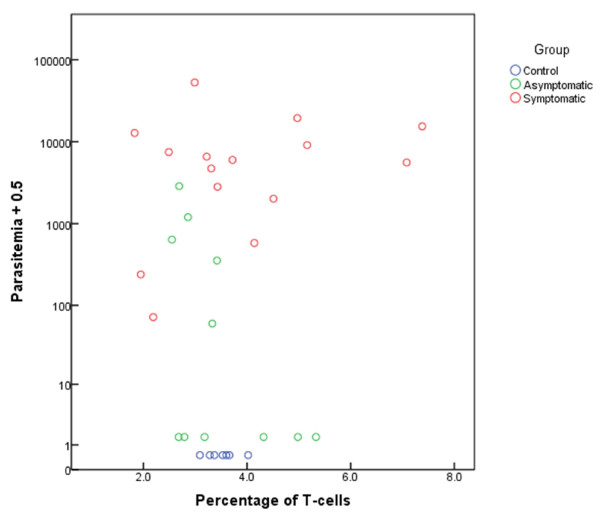
**Correlation of parasitaemia and Tregs.** Variation of blood parasitaemia level for *P. falciparum* according to the percentage of Tregs on control, asymptomatic and asymptomatic subjects.

The percentage of Tregs from S and AS patients was compared with peripheral parasitaemia. No significant linear correlation between Treg percentage and parasitaemia (parasites/ml) was found [r = 0.02, *p* = 0.47] (Figure 2).

## Discussion

This study showed that Symptomatic (S), Asymptomatic (AS) and Control (C) groups had similar absolute numbers and percentage of Tregs in peripheral blood, 3.9%, 3.5% and 3.5% respectively, and a relationship between Treg percentage and parasitaemia was not found (Figures 1 and 2). Therefore, there was no support for the hypothesis that in the *P. falciparum* hypoendemic region of the Peruvian Amazon, absolute or relative numbers of Tregs relates to malaria symptomatology. These results stand in contrast to previous studies in mouse and from humans in other malaria regions [4,9,17-20], where the expansion of Tregs was found to be significantly associated with increased parasite load and development of clinical malaria.

Some studies have compared numbers of Tregs with severe or uncomplicated malaria, and found also increased number of Tregs during convalescence negatively associated with the magnitude of Th1 memory response in children [21], and the increase associated with parasite biomass in severe but not complicated cases in adults [22]. Studying other species, adults with uncomplicated *P. vivax* infections were also found to have increased proportions of CD4^+^ CD25^+^ Foxp3^+^ Treg cells in Thailand [23] and Brazil [24]. A different result was demonstrated by Gonçalves *et al.*[25] who found that malaria patients from hypo- to meso-endemic region had lower absolute numbers of Treg cells than controls related to lower overall CD4^+^ cell counts. In contrast to our observations, this previous study showed that the CD25^+^ Foxp3^+^ Treg cell counts were inversely proportional to level of parasitaemia [26].

These results showed that the absolute numbers and proportion of Tregs were similar among symptomatic and asymptomatic groups despite a higher parasitaemia in S group vs. AS group, confirming that the relationship between number of parasites and Tregs activity is still not clear. Therefore, at least in the context of the hypoendemic setting, total numbers of Tregs are not implicated in the symptomatology or control of parasite proliferation. These results agree with previous reports that showed a more efficient Th1 and Th2 response toward *P. falciparum* antigens [27]. A study carried out in Fulani, a West African ethnic group with low susceptibility to malaria, reported that this population’s PBMCs express higher amounts of RNA for several Th1 and Th2 related genes but gene expression of FOXP3 and cytotoxic T lymphocyte antigen 4 (CTLA4) was markedly lower [28]. These results, compared to other studies, suggest that activity of Tregs in this ethnic group is not involved in regulatory response against malaria.

Although the data presented here did not include analysis of cytokines associated with Tregs, antigen-specific Tregs or possibly a lower expression of molecules involved in Treg cell mediated modulation of immune responses might contribute to the control of symptoms related to symptomatic vs. asymptomatic parasitaemia.

Little is known about characteristics of the malaria parasites themselves associated with the state of asymptomatic infections. A recent study of asymptomatic infected children found an increased tumor necrosis factor receptor type II (TNFRII) expression on Tregs confirming the activation of these cells even in subclinical parasitaemia [29].

The lack of relationship of number or proportion of Tregs to symptomatology in the present study is consistent with previous work where the duration of clinical symptoms and Treg cell number were measured; these studies did not find a significant correlation between these two parameters [30].

The discrepancy among all of these studies, including the current results, may related to differences in study population, study design, Treg phenotypes, age of individuals, cumulative exposure to malaria, clinical outcome of the disease, working conditions, clinical outcome, among others variables.

There are several important limitations of this study. First, pro- and anti-inflammatory cytokines associated with the regulation by Tregs were not measured. The concept that transmission intensity influences the efficiency of acquiring malaria-specific immunological memory has been reported widely. If the majority of infections occur within a sub-population who frequently travel or live in higher transmission regions (i.e. Africa), then these highly exposed individuals might receive the quantity of infections conventionally considered necessary develop protective immunity against symptoms during infection. Conversely, if infections are acquired locally, then there would be low transmission in the whole population and one would expect most of the infections to be symptomatic. This expectation is based upon literature regarding the development of naturally acquired immunity to *P. falciparum* malaria requiring frequent infections over a period of five years and sustained frequent infections in order to maintain an ability to resist symptoms during malaria infection. With these data the function of Tregs might be different in these regions because of different transmission context, intensity and other unknown factors. Another important consideration is that patients with symptomatic parasitaemia might only have had incidental parasitemia along with a separate concomitant infection responsible for causing the fever; we did not assess this possibility in this study.

The present work is the first to report about number and proportion of Tregs in symptomatic *vs.* asymptomatic *P. falciparum* infected individuals from a malaria hypoendemic region, typified by the transmission patterns founds in the Peruvian Amazon region. Deeper phenotypic and functional characterization of Tregs in malaria in this and comparable regions may yield new insights into the immunological control of malaria symptomatology in the face of asymptomatic parasitaemia.

## Competing interests

The authors declare that they have no competing interests.

## Authors’ contributions

KT designed the study, carried out the immunological analysis and wrote the paper. EV and JB performed some experiments and contribute to the paper writing. JCH carried out the statistical analysis. JV and DG supervised the work and study design, wrote and reviewed the final versions of the manuscript. All authors read and approved the final manuscript.
